# Anatomical Characteristics and Biomechanical Properties of the Oblique Popliteal Ligament

**DOI:** 10.1038/srep42698

**Published:** 2017-02-16

**Authors:** Xiang-Dong Wu, Jin-Hui Yu, Tao Zou, Wei Wang, Robert F. LaPrade, Wei Huang, Shan-Quan Sun

**Affiliations:** 1 Department of Orthopaedic Surgery, The First Affiliated Hospital of Chongqing Medical University, Chongqing 400016, China; 2 The First College of Clinical Medicine, Chongqing Medical University, Chongqing, 400016, China; 3 Department of Neurosurgery, The First Affiliated Hospital of Chongqing Medical University, Chongqing 400016, China; 4 Department of Gastroenterology, The First Affiliated Hospital of Chongqing Medical University, Chongqing 400016, China; 5 Department of Obstetrics & Gynecology, The First Affiliated Hospital of Chongqing Medical University, Chongqing 400016, China; 6 The Steadman Clinic, Vail, Colorado 81657, USA; 7 Human Gross Morphology Lab, National Class Preclinical Medicine Experimental Teaching Demonstration Center, Chongqing Medical University, Chongqing 400016, China

## Abstract

This anatomical study sought to investigate the morphological characteristics and biomechanical properties of the oblique popliteal ligament (OPL). Embalmed cadaveric knees were used for the study. The OPL and its surrounding structures were dissected; its morphology was carefully observed, analyzed and measured; its biomechanical properties were investigated. The origins and insertions of the OPL were relatively similar, but its overall shape was variable. The OPL had two origins: one originated from the posterior surface of the posteromedial tibia condyle, merged with fibers from the semimembranosus tendon, the other originated from the posteromedial part of the capsule. The two origins converged and coursed superolaterally, then attached to the fabella or to the tendon of the lateral head of the gastrocnemius and blended with the posterolateral joint capsule. The OPL was classified into Band-shaped, Y-shaped, Z-shaped, Trident-shaped, and Complex-shaped configurations. The mean length, width, and thickness of the OPL were 39.54, 22.59, and 1.44 mm, respectively. When an external rotation torque (18 N·m) was applied both before and after the OPL was sectioned, external rotation increased by 8.4° (*P* = 0.0043) on average. The OPL was found to have a significant role in preventing excessive external rotation and hyperextension of the knee.

The oblique popliteal ligament (OPL) is the largest structure on the posterior aspect of the knee, and given its broad shape, it is probably vulnerable to, or easily involved in, posterior knee injuries^1^. However, current surgical reconstruction procedures usually ignore potential tears of the OPL, thus inducing an inability to reproduce native dynamic knee function^1^,^2^. Insufficient awareness of the anatomic characteristics, radiographic features, and biomechanical properties of the OPL may result in a failure to recognize and replicate normal anatomical structures and functional rehabilitation in the management of traumatic injuries to the knee joint. Thus, a combined anatomical and biomechanical study of the OPL would contribute to “rediscovery” of the posterior structures and corresponding function of the knee, thereby providing a more anatomical and functional basis for the treatment of OPL injuries.

Several investigators have attempted to explore the characteristics of the OPL^1^,^2^,^3^,^4^,^5^. However, descriptions of the morphology and role of the OPL in the knee joint remain inconsistent in the literature. Some textbooks and articles^3^,^4^,^5^ report that the OPL originates with the tendon of the semimembranosus muscle, whereas other sources have described that the OPL is formed by a confluence of the lateral expansion of the semimembranosus tendon and the capsular arm of the posterior oblique ligament^1^,^6^,^7^. The course of the OPL is fairly similar, and its terminal attachments are relatively uniform, but the description of its arms or attachments is variable. Furthermore, there remains a paucity of data describing the external features, anatomical characteristics, and biomechanical properties of the OPL.

It has also been reported that the OPL is a primary ligament restraint to knee hyperextension^2^,^8^. However, on the basis of the oblique course of the OPL and the Parallelogram of Force (Fig. 1)^9^, we deduce that the OPL not only has the function of preventing hyperextension, but also plays a significant role in preventing excessive external knee rotation. Therefore, given the contradictions and limitations found in the literature, it is necessary to perform further study of the anatomical and biomechanical characteristics of the OPL, which would contribute to the recognition and restoration of OPL injuries and would help surgeons avoid iatrogenic injuries.

The purpose of the present study was twofold. First, this study attempted to provide a detailed description of the anatomical structures and to complement the quantitative data on the OPL in the Asian population. Second, our study sought to characterize the biomechanical properties and to provide a comprehensive analysis of the primary function of the OPL.

## Results

### Anatomy of the OPL

**Figure 1:**
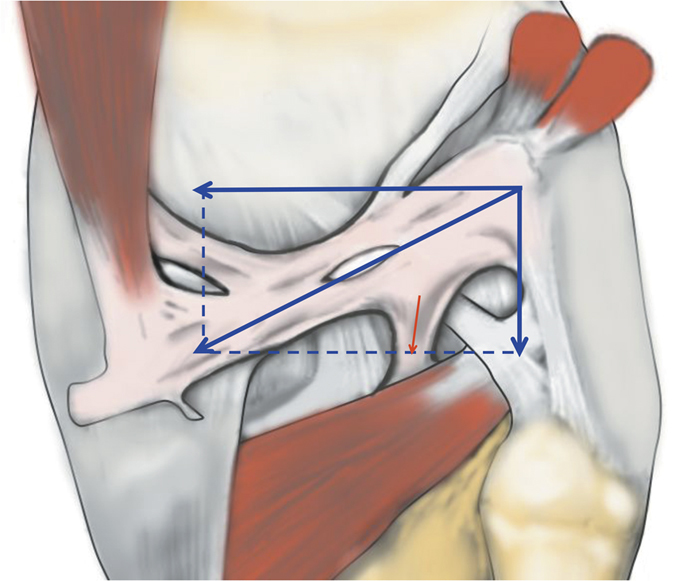
Illustration of the Parallelogram of Force applied in the oblique popliteal ligament.

The OPL was identified in all specimens as a broad and flat ligament that diagonally crossed the posterior knee joint and overlaid the posterior surface of the knee joint capsule. The OPL was a well-defined ligament that crossed superficially from the posteromedial aspect of the capsule and attached to the posterolateral corner of the knee. In each knee pair in our study, the morphology of the OPL was roughly symmetrical bilaterally. The OPL originated from the posterior surface of the posteromedial tibial plateau and blended with fibers from the semimembranosus tendon (Fig. 1). Additionally, some fibers from the posteromedial part of the capsule blended with the OPL and formed a secondary medial origin. The two origins merged and coursed superolaterally, then attached to the fabella or to the tendon of the lateral head of the gastrocnemius (if there was no bony fabella), blending into the posterior capsule over the lateral femoral condyle.

Additionally, the middle genicular neurovascular bundle entered the midsection of the OPL and passed through elliptical-shaped holes in the posterior joint capsule to supply the cruciate ligaments and synovial membrane (Fig. 1). In our specimens, 11 pairs of 15 had two obvious holes, and the other four pairs had one hole. The shapes of the holes varied from ellipsoid to a long ellipse. The major axes of the elliptic holes were parallel with the courses of the OPL. Different shapes and entry points of the neurovascular holes divided the OPL into two (in 10 pairs of 15) or three (in 5 pairs of 15) bundles.

The origins and final insertions of the OPL were relatively stable, but there was significant variability in the attachments and shapes of the OPL. The most frequent branch was the tibial expansion of the OPL. The tibial expansion originated from the posterolateral corner of the knee and ran almost vertically downward, then attached to the tibia. The tibial expansion of the OPL was palpable in 7 pairs of 15, some of which were thin (2 pairs of 7), but most of which were thick and tough (5 pairs of 7). At the middle and distal part of the OPL, there were sparse fibers that attached at the upper margin of the intercondylar fossa and the posterior surface of the femur. The insertion of the OPL also blended with the fibers of the fabellofibular ligament.

**Figure 2:**
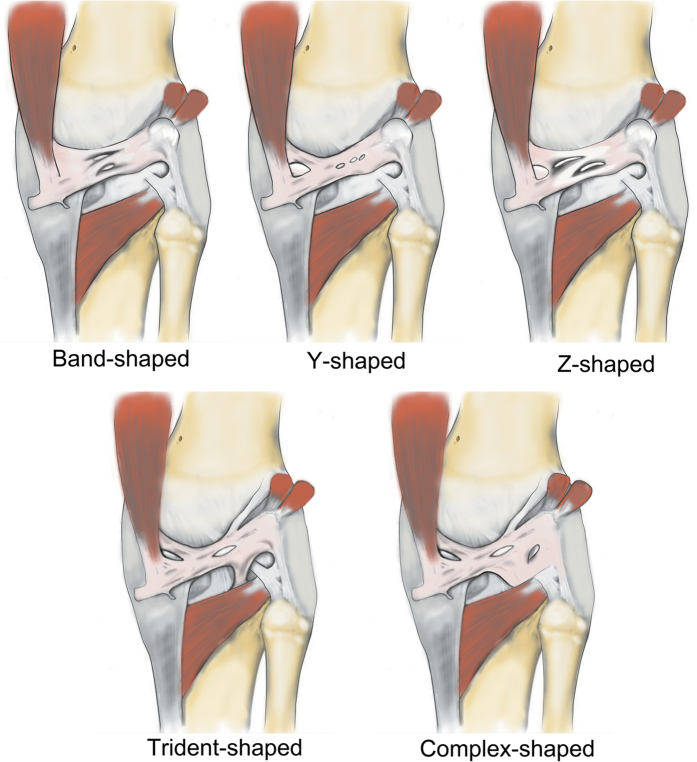
Illustration of five different patterns (Band-shaped, Y-shaped, Z-shaped, Trident-shaped, and Complex-shaped) of the oblique popliteal ligament.

**Figure 3:**
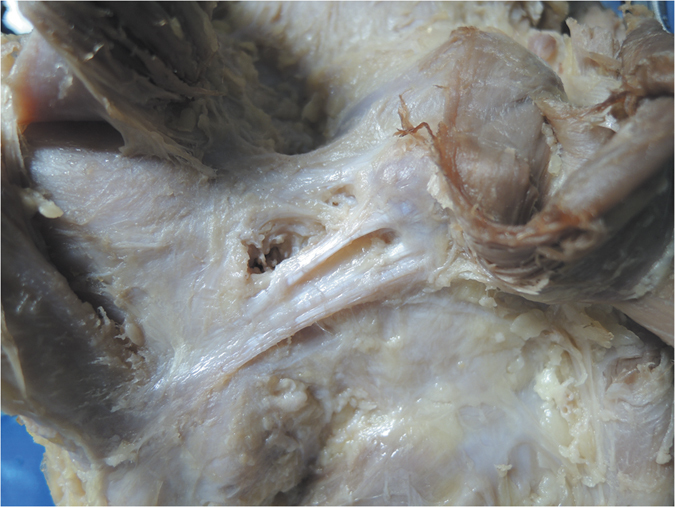
Anatomical photograph of the Band-shaped oblique popliteal ligament (posterior aspect of the right knee).

**Figure 4:**
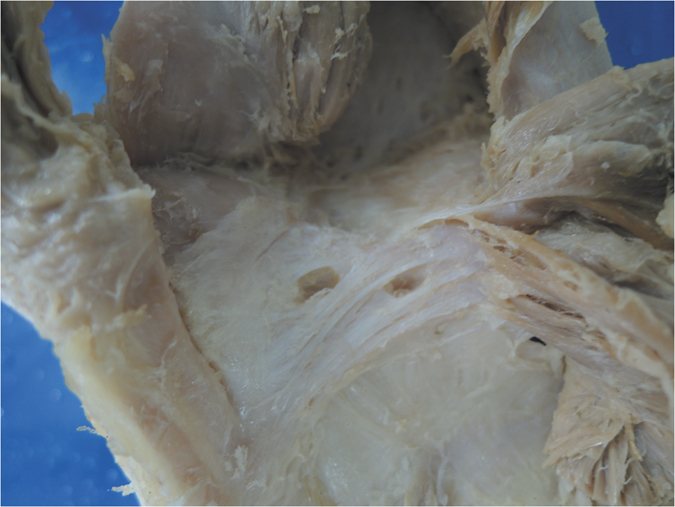
Anatomical photograph of the Y-shaped oblique popliteal ligament (posterior aspect of the right knee).

**Figure 5:**
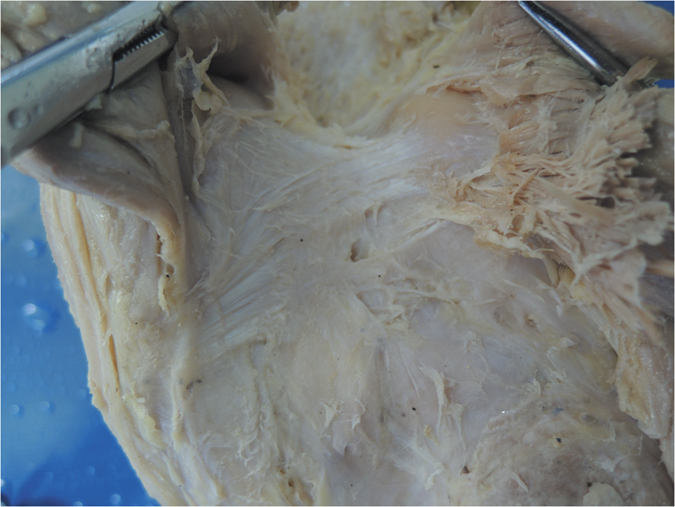
Anatomical photograph of the Y-shaped oblique popliteal ligament (posterior aspect of the right knee).

**Figure 6:**
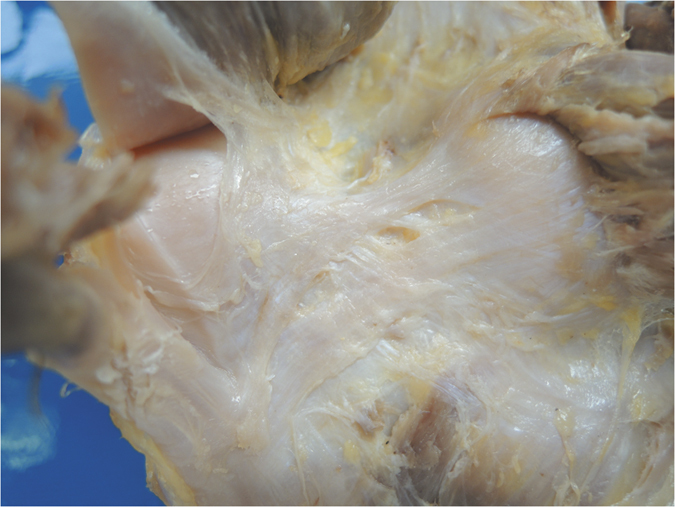
Anatomical photograph of the Y-shaped oblique popliteal ligament (posterior aspect of the right knee).

**Figure 7:**
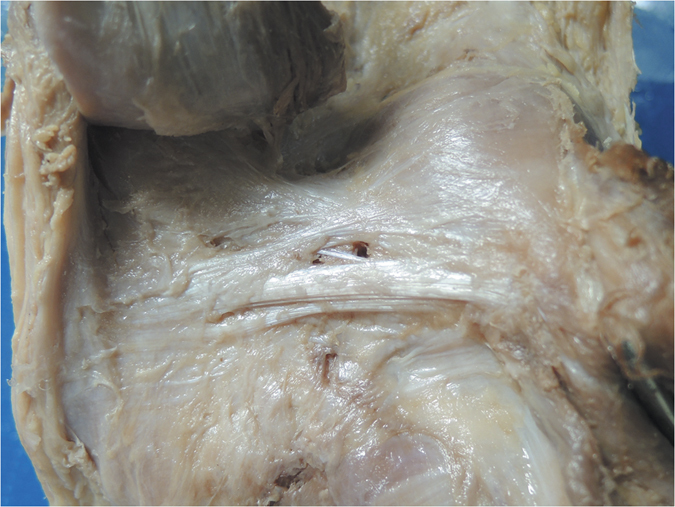
Anatomical photograph of the Z-shaped oblique popliteal ligament (posterior aspect of the right knee).

**Figure 8:**
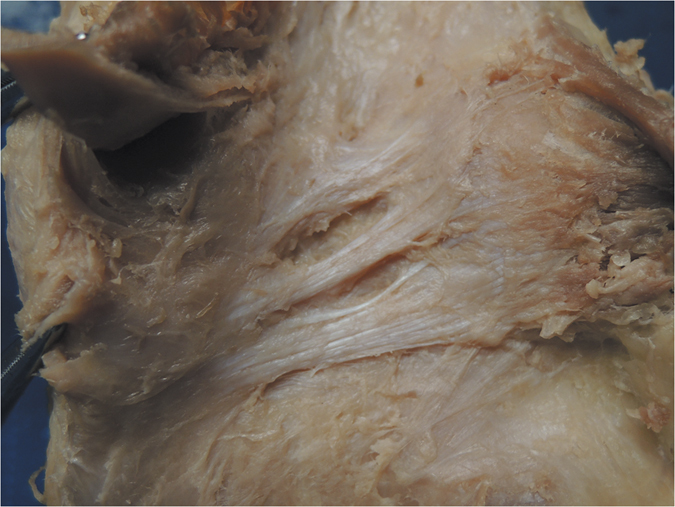
Anatomical photograph of the Z-shaped oblique popliteal ligament (posterior aspect of the right knee).

**Figure 9:**
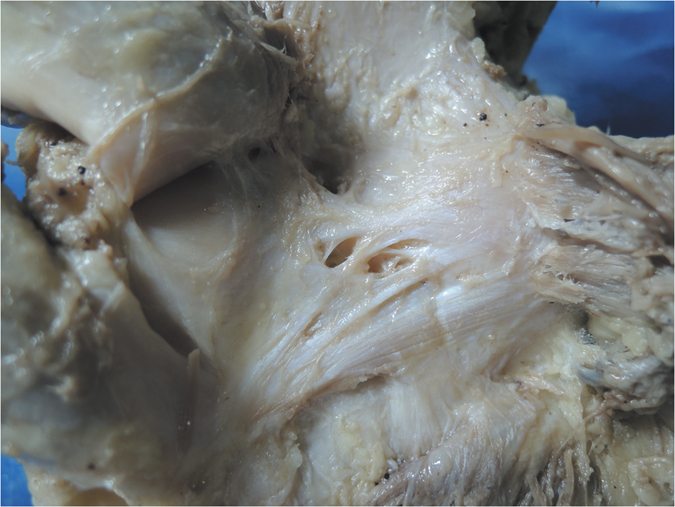
Anatomical photograph of the Trident-shaped oblique popliteal ligament (posterior aspect of the right knee).

**Figure 10:**
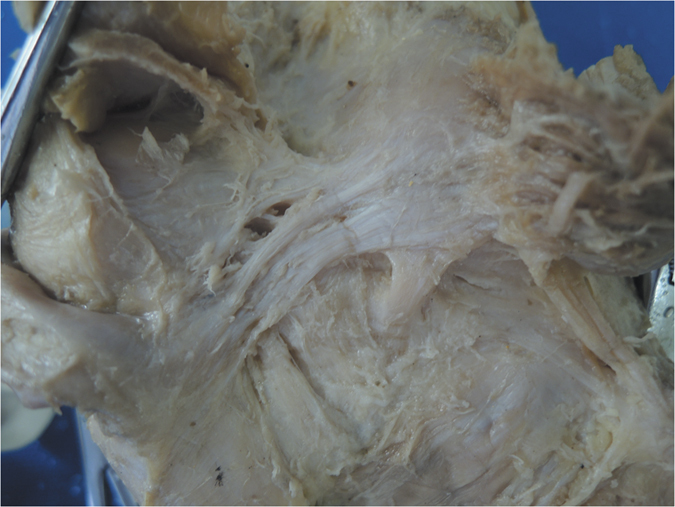
Anatomical photograph of the Trident-shaped oblique popliteal ligament (posterior aspect of the right knee).

**Figure 11:**
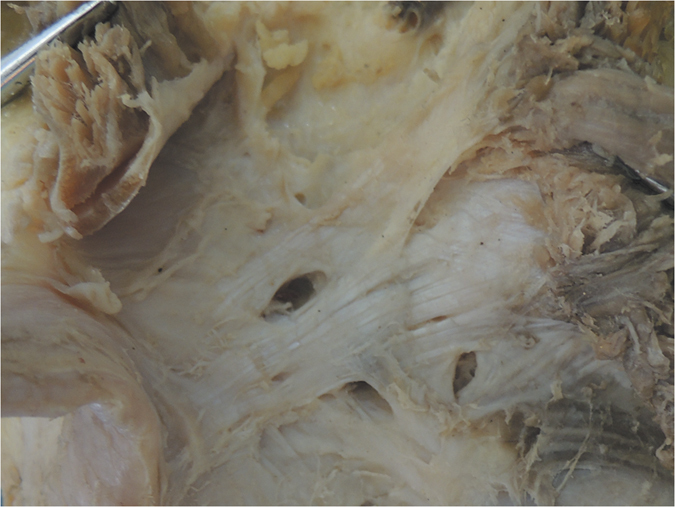
Anatomical photograph of the Complex-shaped oblique popliteal ligament (posterior aspect of the right knee).

Furthermore, we classified the OPL into five types according to the current external features of the OPL (Fig. 2): Band-shaped (5 pairs), Y-shaped (4 pairs), Trident-shaped (3 pairs), Z-shaped (2 pairs), and Complex-shaped (1 pair). The Band-shaped OPL did not have attachments to the tibia, and the two origins did not have an obvious separation; thus, it was stripe-shaped like a band (Figs 2 and 3). The Y-shaped OPL had two obvious separate origins: one originating from the medial tibial plateau and blending with semimembranosus tendon, and the other originating from the posteromedial part of the capsule (Figs 2, 4, 5 and 6). The Z-shaped OPL obtained its name mainly on the basis of two slender ellipses parallel to the three separate bundles (Figs 2, 7 and 8). The Trident-shaped OPL had two distinct origins and one main central tibial expansion of the OPL (Figs 2, 9 and 10). Finally, the Complex-shaped OPL was relatively uncommon and included double tibial expansions, sparse fibers attached to the margin of the intercondylar fossa, and a fiber bundle to the tendon of the plantaris muscle (Figs 2 and 11).

The mean length of the OPL was 39.54 ± 4.64 mm, but the ligament width was more variable. The average width at the midpoint was 22.59 ± 5.09 mm at the midpoint, and the average thickness at the midpoint was 1.44 ± 0.40 mm. The mean angle between the OPL and the joint line was 29.78 ± 2.62°.

### Biomechanical properties

When we performed the hyperextension experiment, we observed that the OPL and tibial expansion of the OPL became tensioned, thus indicating its function in preventing knee hyperextension. When the flexion angle was set to 45° and a rotational torque was applied on the tibia, the tibia externally rotated and the OPL was qualitatively observed to be taut. The intact state external rotation angle averaged 20.2°; after the OPL was sectioned and the same rotational torque was applied, the average increase in tibial external rotation was 8.4° (*P* = 0.0043).

## Discussion

The purpose of this descriptive laboratory study was to explore the anatomical characteristics and biomechanical properties of the OPL. To the best of our knowledge, this is the first study to date to describe the morphology of the OPL in detail and to classify the shape of the OPL into different types. Furthermore, we also performed a biomechanical analysis to demonstrate that the OPL plays significant roles in preventing both excessive external knee rotation and knee hyperextension, thus validating the structural properties of the OPL in providing stability to the human knee. Notably, this is also the first study to report on the size parameters of the OPL in the Chinese population.

The anatomic characteristics and biomechanical properties of the OPL have been recognized for a long time^10^,^11^,^12^,^13^. However, with the rapid development of sports medicine, the key stabilizing elements of the knee have recently been rediscovered^1^,^2^,^4^,^14^,^15^,^16^,^17^. Customarily, these studies have dissected and described the anatomy of fresh-frozen human cadaveric knees and conducted quantitative biomechanical experiments^1^,^2^,^3^,^4^,^18^,^19^,^20^,^21^. Although these explorations^1^,^2^,^3^,^5^ have described the shape and parameters of the OPL and have provided quantitative biomechanics, which have contributed to a better understanding of the role of the OPL in preventing knee hyperextension^2^, the diversity of the morphology of the OPL has rarely been reported to date, and its function of preventing excessive external rotation of the knee was neglected in studies.

It is unknown whether prenatal heredity, postnatal development, or both cause the various OPL shapes. We infer that the different types of OPL would produce slight differences in knee stability when they are injured. Compared with the Band-shaped OPL, the origin of the Y-shaped OPL was divaricate, thereby contributing to resolving the bearing strength of the OPL. Compared with the Y-shaped OPL, the most prominent characteristic of the Z-shaped OPL was its division into bundles, thus reciprocally spreading the force among the bundle bands. The Trident-shaped and Complex-shaped OPL had multiple attachments and arms, thus making it easier to spread force to the surrounding structures; the unfavorable consequence of such a divaricate structure is that it may be more vulnerable and easily damaged or involved in peripheral injuries. Additional research is warranted to explore exactly how the force is resolved from the trunk of the ligament to these multiple insertions or attachments. Such knowledge would contribute to understanding and predicting the individual impaired segments.

The average length of the OPL in our study was approximately 39.54 ± 4.64 mm, which was shorter than the 48.0 mm (range, 43.0–55.0) reported by LaPrade *et al*.^1^ and 45.56 ± 4.67 mm reported by Osti *et al*.^4^, but longer than the 33.6 ± 4.8 mm reported by Fam *et al*.^3^. The mean angle between the OPL and the horizontal joint line in our study was 29.78 ± 2.62°, which was smaller than the 32.2 ± 6.6° reported by Fam *et al*.^3^. These differences may be primarily attributed to different methods of measurement; another potential explanation is ethnic differences, although this possibility is uncertain, owing to limited data.

The OPL is an important stabilizing structure on the posterior aspect of the knee joint, and it also provides an important reinforcing function in joint stability. However, its biomechanical characteristics together with its structural properties, have not been well recognized. Although a previous study has reported its role in preventing knee hyperextension^11^, a recent in-depth study applying a sequential sectioning technique on the major structures of the posterior knee has found that the OPL is the primary ligamentous restraint to knee hypertension, thus highlighting the biomechanical importance of the OPL^2^. Similarly, both the “posterolateral corner” and the “posteromedial corner” resist tibial rotation^22^, yet a main primary stabilizer against excessive external knee rotation has not been well identified and requires further exploration.

Because of the diagonal oblique course of the OPL, the forces along it could be separated into a horizontal force and a vertical force (Fig. 1); it is therefore logical, that the horizontal force can prevent excessive external rotation and the vertical force can prevent hyperextension. The horizontal force was greater than the vertical force according to the angle down from the horizontal joint line, thus possibly indicating that preventing excessive external knee rotation is a relatively more important function of this structure. In extreme cases (Figs 6 and 7), the OPL was horizontally located in the posterior aspect of the knee, thus highlighting its unique function in preventing excessive external knee rotation. Moreover, the neurovascular apertures on the OPL were generally elliptical, and the long shaft paralleled the course of the main OPL trunk, a result that may indicate flexible margins of the OPL. Thus, the biomechanical function of the OPL appears to be preventing both excessive external knee rotation and knee hyperextension. When the knee joint moves from flexion to extension, there would be an accompanied tibial axial external rotation^23^,^24^,^25^,^26^, and the OPL provides stability in hyperextension and axial rotation in normal activities, extreme sports, and serious trauma.

The knee is one of the largest and most complex pivotal hinge joints in the body. The patella and patellar ligament and four key ligaments of the knee including the anterior/posterior cruciate ligaments and the medial/lateral (fibular) collateral ligaments are responsible for the anterior, medial, and lateral stability of the knee joint; however, the posterior aspect of the knee is relatively feeble. Another important function of the OPL is to reinforce the posterior knee capsule, which together with other ligaments forms a circumferential sleeve around the knee, thus providing stability to the knee overall. Even so, there remains an area without external support between the OPL, the expansion over the popliteus muscle, and the lateral side of the posterior cruciate ligament, which are strongly related to the formation of popliteal cysts^27^,^28^,^29^. The elliptical holes in the OPL (Figs 3,4,7 and 8) are also weak areas and may be associated with popliteal cyst formation^30^. Therefore, the morphology and attachments of the OPL may influence the formation of popliteal cysts to some extent.

The tibial expansion of the OPL that belongs to the posterolateral corner of the knee has been reported to strengthen the knee in hyperextension^31^. Attention should be focused on preventing iatrogenic injuries to the OPL, especially to the tibial expansion, during a posterior approach to the knee^32^. Damage to the OPL or the tibial expansion of the OPL can be debilitating to patients and may require prompt recognition and treatment to avoid long-term consequences^33^. If there is posterolateral instability of the knee or in planning for surgical intervention, the involvement and biomechanical function of the OPL should be considered.

Injuries to the posterior aspect of the knee, especially the posterolateral corner, are common^21^,^34^. It has not been well studied whether the OPL attachments are involved in these injuries. Magnetic resonance imaging (MRI) scans should be recommended to evaluate injury to the OPL or its attachments^35^,^36^,^37^,^38^,^39^.

### Limitations

Our study has several limitations. First, because it was difficult to obtain fresh-frozen cadaveric knees in our institute, we had to utilize specimens fixed with formalin in our experiment. This issue probably had no effect on the description of the shape of the OPL and its classification; however, the biomechanical testing data may have been strongly influenced. Nonetheless, as mentioned previously, we were still able to demonstrate its function in preventing excessive external knee rotation and hyperextension. Second, the self-designed tensile testing instrument did not have a high-precision measurement tool; however, it was adequate for this experiment.

## Conclusion

The medial and lateral attachments of the OPL were relatively consistent, but its course and structure were variable and were classified into types on the basis of its shape. The OPL plays significant roles in preventing both excessive external knee rotation and hyperextension and in strengthening the stability of the knee. Improved recognition of the OPL may help define its involvement in posterior knee injuries and the consequences of damaging this ligament during posterior knee surgery.

## Materials and Methods

### Specimen information

This descriptive laboratory study used 15 pairs of embalmed cadaveric knee specimens from 11 male and 4 female Chinese donors. Knees with damaged posterior structures, a history of injury or surgery, cachexia, ligamentous injury, or indications of osteoarthritis were excluded. The average age of the donors at the time of death was 62.7 years (range, 49–76). This study was approved by the Institutional Review Board of Chongqing Medical University. All methods and procedures were performed in accordance with the relevant guidelines and regulations^40^. Informed consent was obtained from all subjects.

### Anatomy dissection

Preparation, detailed dissection, and sectioning of the knees were performed according to a standardized procedure. The dissection was performed by means of posterior access. A gross anatomic dissection was performed to completely remove the skin and subcutaneous tissues around the knee. Next, precise dissection of the posterior structures of the knee was performed to identify the deep layers. We removed the neurovascular bundle to ensure sufficient exposure and to improve visualization. The dissections consisted of identification of the deep layer of the knee which was located between the posterior borders of the semimembranosus and the tibial course of the superficial medial collateral ligament medially, and the medial border of the long head of the biceps femoris and fibula laterally. All relative structures anterior (deep) to the medial and lateral heads of the gastrocnemius and semimembranosus were preserved and identified. The OPL was then isolated and cleared of residual tissues, the origins were dissected, and the attachments were identified. The OPL and the structures confluent with it were also dissected, carefully observed, recorded, and measured. Additionally, the morphology of the OPL was classified into types according to the external features on the basis of its shape.

### Quantitative analysis

Characteristics of the OPL and its orientation relative to the surrounding structures were recorded after OPL identification. The size of the OPL was measured to provide supplemental data about the OPL in the Chinese population. To perform the measurements, needles were used to identify the precise origin and insertion points and bony landmarks, and then the OPL was measured with a Vernier caliper with a reported measurement accuracy to 0.02 mm (JiangHua, Shanghai, China). The length was measured by taking the distance from the origin of the major structures of the OPL to the selected osseous landmarks (the midportion of the capsule on the femur distally). The width and thickness of the OPL were gauged at the midpoint of the OPL. The angle between the OPL and the horizontal joint line was measured with Image-Pro Plus version 6.0 (Media Cybernetics, Silver Spring, MD, USA). The measured data were reported as the means with standard deviations (SD), which were calculated using the appropriate *t*-score with SAS software, version 8.0 (SAS Institute, Cary, NC, USA).

### Biomechanical testing

**Figure 12:**
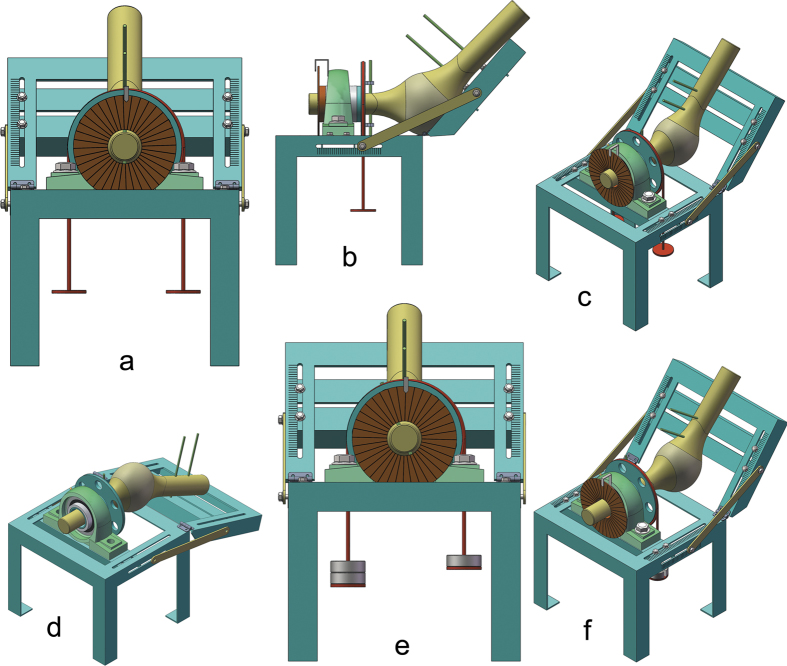
The diagram of our self-designed testing apparatus for biomechanical tensile testing. (**a**) Illustration of our self-designed testing apparatus (front view): the circular scale (measurement accuracy 0.1°) with a pointer was fixed at the terminal of the tibia and rotated together with the tibia; (**a**) large-diameter lightweight sheave was wrapped around for several coils with (**a**) strap, and each end of the strap was tied to unequal weights that generated a constant vertical downward torque. (**b**) Illustration of our self-designed testing apparatus (lateral view): the large-diameter lightweight sheave was bound and rotated together with the tibia, and the sheave transmitted the torque to the tibia simultaneously; the femur was fixed on the mounting bracket; and the tibia rotated in the sleeve. (**c**) Illustration of our self-designed testing apparatus (oblique view): the angle between the femur and tibia could be regulated. (**d**) Illustration of verifying the restraint effect of hyperextension: the posterior knee was upward, and we applied a hyperextension force to qualitatively observe and verify its function. (**e**) Illustration of testing the effect of preventing excessive external knee rotation: the strap was tied to unequal weights and generated a constant vertical downward torque. (**f**) Illustration of testing the effect of preventing excessive external knee rotation: the tibia appeared to rotate counterclockwise, and the rotation angel before and after sectioning the oblique popliteal ligament was measured and recorded.

Biomechanical characteristics of the OPL were tested using a self-designed tensile testing instrument (Fig. 12a–c). The instrument was composed of three parts: a mounting bracket, a torsion apparatus, and a circular scale (measurement accuracy 0.1°) with a pointer. The mounting bracket provided fixation of the femur and tibia and was simultaneously used to regulate the angle between the femur and tibia. The torsion apparatus was mainly used to apply torque (measurement accuracy 0.5 N·m) on the tibia, with the torque arising from a pair of unequal and equidirectional forces. A large-diameter lightweight sheave was bound and rotated together with the tibia; another strap was wrapped around the sheave for several coils; and each end of the strap was tied to unequal weights, thus generating a constant vertical downward torque. The sheave transmitted the torque to the tibia simultaneously. The circular scale fixed at the terminus of the tibia also rotated together with the tibia, and a stationary pointer reported the rotation angle. To verify the restraint effect of hyperextension, we applied a hyperextension force (Fig. 12d) and to qualitatively observe its function and confirm the results of previous studies^2^. To determine whether the OPL plays a role in preventing excessive external knee rotation, before and after sectioning was performed, we rigidly anchored the distal end of the femur and set the flexion angle to 45° (Fig. 12e,f). A rotational torque (18 N·m) was applied to the tibia to induce external rotation of the lower limb, and the rotation angle was measured and recorded. The OPL was then sectioned; the same rotational torque was applied; and the rotation angle was measured and recorded again. All anatomical and biomechanical measurements are reported as mean values.

## Additional Information

**How to cite this article**: Wu, X.-D. *et al*. Anatomical Characteristics and Biomechanical Properties of the Oblique Popliteal Ligament. *Sci. Rep.* **7**, 42698; doi: 10.1038/srep42698 (2017).

**Publisher's note:** Springer Nature remains neutral with regard to jurisdictional claims in published maps and institutional affiliations.
