# Corrigendum: Anatomical Characteristics and Biomechanical Properties of the Oblique Popliteal Ligament

**DOI:** 10.1038/srep46117

**Published:** 2017-04-10

**Authors:** Xiang-Dong Wu, Jin-Hui Yu, Tao Zou, Wei Wang, Robert F. LaPrade, Wei Huang, Shan-Quan Sun

Scientific Reports 7: Article number: 4269810.1038/srep42698; published online: 02 16 2017; updated: 04 10 2017

This Article contains an error in the Discussion section.

“Even so, there remains an area without external support between the OPL, the expansion over the popliteus muscle, and the lateral side of the posterior cruciate ligament, which are strongly related to the formation of popliteal cysts”.

should read:

“Even so, there remains an area without external support between the OPL, the expansion over the popliteus muscle, and the medial side of the posterior cruciate ligament, which are strongly related to the formation of popliteal cysts”.

